# Dietary resveratrol improves immunity but reduces reproduction of broodstock medaka *Oryzias latipes* (Temminck & Schlegel)

**DOI:** 10.1007/s10695-016-0265-8

**Published:** 2016-07-18

**Authors:** Agata Kowalska, Andrzej K. Siwicki, Radosław K. Kowalski

**Affiliations:** 1 Department of Aquaculture, The Stanisław Sakowicz Inland Fisheries Institute, Oczapowskiego 10, 10-719 Olsztyn, Poland; 2 Department of Microbiology and Clinical Immunology, Faculty of Veterinary Medicine, University of Warmia and Mazury, Oczapowskiego 13, 10-719 Olsztyn, Poland; 3 Department of Gamete and Embryo Biology, Institute of Animal Reproduction and Food Research, Polish Academy of Sciences, Tuwima 10, 10-748 Olsztyn, Poland

**Keywords:** Resveratrol, Immunomodulators, Sperm, Egg, Fish

## Abstract

Here, we investigated the effect of dietary resveratrol (20, 40, and 80 µg/g BW/day) on cell-mediated immunity (activity of spleen phagocytes and proliferative response of lymphocytes) and reproductive parameters (egg and sperm quality, i.e. fecundity—total number of eggs produced by individual fish, fertility, embryo survival, and hatching rate) in medaka. Fish fed feed with resveratrol at 40 and 80 µg/g BW/day had significantly higher metabolic activity and intracellular phagocyte killing activity than control. The proliferative lymphocyte activity of the fish from R80 group was greater by more than 20 % in comparison with the control group (*P* < 0.05). The percentage of macrophages (MO) and their mean fluorescence intensities (MFI) in R40 and R80 groups were significantly higher compared to C and R20 groups (*P* < 0.05). The differences in MO and MFI values ranged from 52.5 % (±1.5; R0 group) to 65.8 % (±1.6; R80 group) and from 23.2 (±1.4; R0 group) to 38.2 (±2.4; R80 group), respectively. Moreover, resveratrol at 80 µg/g BW/day decreased liver COX activity, i.e. 5.4 in R80 group and 7.9 in R0 group (*P* < 0.05). The motility parameters of the sperm obtained from the males fed feed supplemented with resveratrol at 80 µg/g BW/day exhibited the highest values except the linearity, which was lower as compared to the control (*P* < 0.05). The results indicate that diet supplemented with resveratrol at a dosage of 40 µg/g BW/day improves phagocyte killing ability and lymphocyte proliferation in broodstock and accelerates offspring hatch. Also, the results suggest that COX activity influences sperm and oocyte quality in fish; the presence of a COX inhibitor in the dose of 40 µg/g BW/day decreased the embryo survival.

## Introduction

As aquaculture expands, research needs to maintain a careful balance between intensive production and the health and disease resistance of fish (Ashley 2007). Reduced resistance and increased susceptibility to disease result from the immunosuppressive effects of physical stress. In addition, some immunosuppressive factors also reduce reproductive performance (Barton and Iwama 1991; Wedemeyer 1997). Feed containing the appropriate quantities of high-quality nutrients helps maintain disease resistance in fish. And certain feed additives are known to enhance the fish immune system (Magnadóttir 2006). Therefore, with the development of feeding strategies that increase production, special care should be taken to maintain or even enhance the immune system of fish.

Medaka *Oryzias latipes* is a popular aquatic model organism. A constant long photoperiod and a stable temperature of 26 °C induce spawning within 1 h of the onset of light. Once reproduction has begun, medaka reproduce daily, laying transparent eggs which are easy to count and observe, as they are deposited under the female tail. Therefore, medaka is an ideal model species for research related to reproduction (Kinoshita et al. 2009).

In recent years, many immunomodulators (e.g. herbal remedies, glucans, yeast extracts) that elevate the activity of components of the immune system of fish (Oliva-Teles 2012) have been tested in aquaculture. Nonetheless, there are limited data available on the effects of resveratrol on the fish immune system and fish reproductive parameters. Resveratrol, a natural phytoalexin, exerts a variety of biological effects (Valenzano et al. 2006), including changes in the function of immune system (Flippin et al. 2007) and protection of hepatocytes against stress (Sahin et al. 2012). This pharmaceutical product also affects the activity of cyclooxygenase enzymes (COX), which are responsible for the conversion of arachidonic acid (ARA) to eicosanoids, i.e. prostaglandins (PGs) (Liu et al. 2006; Furne et al. 2013; Chen et al. 2014). PGs are highly active biologically and play important roles in both fish immunity (Siwicki et al. 1994; Rowley et al. 1995; Hata and Breyer 2004) and reproduction (Hong et al. 2007; Stacey and Goetz 1982).

In fish, two COX genes, responsible for the synthesis of COX1 and COX2, have been identified (Ishikawa and Herschman 2007; Ishikawa et al. 2007). The expression of the COX2 gene is dependent of ARA availability (Furne et al. 2013), although a positive self-regulatory effect of prostaglandin 2 (PGE2) on COX expression has also been reported (Minghetti et al. 1997). Japanese medaka has both COX1 (constitutive) and COX2 (inducible) enzymes, as well as their inhibitors, which effectively block ovulation (Fujimori et al. 2011) and embryo survival (Kowalska et al. 2011). Previously, we demonstrated that addition of arachidonic acid to the diet of medaka increased the fish’s reproductive parameters, but not if a low dose of resveratrol was also given (Kowalska and Kowalski 2014). Resveratrol affects ARA mobilization and COX2 induction in murine macrophages (Martinez and Moreno 2000) and inhibits the production of PGE2 by kidney leukocytes in fish (Castro et al. 2008). However, the specific effects of selective inhibition of COX on the broodstock reproductive and immunological parameters are not known yet.

Here, we evaluated the effect of a diet supplemented with resveratrol on cell-mediated immunity (activity of spleen phagocytes and proliferative response of lymphocytes) and reproductive parameters (egg and sperm quality, i.e. fecundity—total number of eggs produced by individual fish, fertility, embryo survival, and hatching rate) in medaka.

## Materials and methods

### Medaka culture

Medaka broodstock (inbred Hd-rR strain) were obtained from laboratory at the Inland Fisheries Institute (Olsztyn, Poland) from eggs originally stocked at the National Institute of Natural Science (Japan). Fish were reared in accord with the recommended culture conditions for this species and were fed a commercial feed (TetraMin, Germany) (Kinoshita et al. 2009). Test animals (96 males and 108 females; body weight, BW = 0.49 ± 0.03 g; total length, TL = 3.39 ± 0.20 mm) were selected from the broodstock (at 9 months post-hatch) and placed in 12 glass tanks (sex ratio 8♂:9♀ per tank). Each tank (30 cm × 40 cm × 30 cm, 20 l water volume) was equipped with a heater (AQ 25, Aquael, Warsaw, Poland) and an external light (Palm Light, AZOO, California, USA). To obtain fertilized eggs, males and females were kept under a 14 h light (650 lux)/10 h dark photoperiod regime at 26.0 °C, ±0.17 °C which induced spawning. Fish were reared in Iwamatsu’s balanced salt solution (Yamamoto 1975) made with distillated water. Feces were siphoned off daily, and 1/4 of the water in each tank was replaced daily with freshly prepared water. The dissolved oxygen and water pH were >5.5 ± 0.4 mg O_2_/l and 6.0–6.5, respectively. Ammonia and nitrite were generally undetectable. No mortality or specific abnormality was found in the experimental groups. Spawners were kept for 3 weeks.

### Medaka feeding

To gain insight into the effect of resveratrol on broodstock cell-mediated immunity, the effects of different levels of resveratrol added to the diet were examined on the metabolic ability of phagocytes (RBA), the potential killing activity of phagocytes (PKA), lymphocyte proliferation, phagocytic splenocytes (%), and the fluorescence intensity of macrophages. Liver histology, cyclooxygenase activity (COX), and reproductive parameters (gametes and embryo quantity and quality) were also examined.

The fish were divided into four feeding treatment groups, in three replicates. Experimental feeding of the broodstock was carried out over 3 weeks, and the fish were fed at 4 % of BW (Kinoshita et al. 2009) twice daily (1000 and 1500 h at the light condition). For the first week, all fish were fed TetraMin, the recommended commercial diet for medaka. For the second and third weeks, the fish were fed the commercial diet supplemented with resveratrol (≥98 %, Sigma-Aldrich, St. Louis, USA) at 20 (R20 group), 40 (R40 group), and 80 (R80 group) µg/g BW/day. The resveratrol was administered with dry food. The resveratrol was dissolved in ethanol and stored as a stock solution in the dark at 4 °C. The same stock solution was used to prepare feed for all groups. To achieve same volume of resveratrol solution administrated in all group, stock solution was additionally diluted with ethanol (2 times for R40 group and 4 times for R20 group). Resveratrol solution was sprayed into Petri dish (on dry food) and immediately mixed with the dry food. The control group of fish (R0 group) was fed the commercial diet mixed as above but with only the ethanol solvent administrated in the same volume as for treatment groups. After it had been mixed, the food was kept for 3 h at room temperature to allow the ethanol to evaporate. Feed was prepared daily.

### Embryo collection and incubation

Spawning occurred usually about 2 h after the start of the light period. Spawned eggs were collected daily from the abdomen of each female approximately 3 h after the start of the light period. The embryos were maintained in Petri dishes (60 mm in diameter) filled with embryo culture medium (100 ppm NaCl, 30 ppm KCl, 160 ppm MgSO_4_·7H_2_O, 40 ppm CaCl_2_·2H_2_O) and ethylene blue (0.5 ppm) as an antifungal prophylactic (Kinoshita et al. 2009). Stocking density was up to 50 eggs/dish (from 30 ± 5.9 in R20 group to 40 ± 8.9 in R40 group), and the temperature ranged from 23.3 to 23.5 °C with differences of less than ±0.23 °C between dietary treatments. The number of fertilized eggs and average egg size (±0.01 mm; NIS-Elements Br version 3.2) were recorded. After 24 h, non-viable eggs were removed and the water was changed. The embryos were observed using a stereoscopic microscope (Nikon SMZ-10A, Tokyo, Japan). Any dead embryos were removed and counted to determine the survival rate of embryos. The fertilization rate was calculated as the average value for all spawned females from each aquarium. The survival of embryos and hatching rates were calculated for embryos and hatches produced in the first, second, and third weeks of broodstock rearing.

Hatched larvae were placed into tanks according to the dietary treatment of their broodstock and were reared in the same manner as for juveniles (Kinoshita et al. 2009) until sexual maturity, which was attained at 10 weeks. After fish attained sexual maturity, each fish was examined to determine its sex (body pigmentation and fin morphology).

## Experimental procedure

### Sampling liver, spleen, and testis tissues

The liver (from females), spleen (from females and males), and testis were dissected and collected for immunological, biochemical, and histological analysis. After the three-week broodstock rearing period, all fish were anesthetized on water ice bath for 1 min and subsequently killed by dissecting of spinal cord. All experimental procedures were accepted by the local ethical commission. The females were measured (BW; ±0.01 g), and their livers were excised and weighed (LW; ±0.001 g) to quantify the hepatosomatic index (HSI = 100 × LW/BW; *N* = 9; three fish/tank; *N* = 3). Then, the livers were processed for histological analysis (*N* = 9; three fish/tank; *N* = 3). The livers for COX enzyme activity analysis were obtained from the remainder of the females (*N* = 15; five fish/tank; *N* = 3). The spleens from 45 individuals from each of the experimental groups were separated for immunological study (*N* = 45; 15 fish/tank; *N* = 3). The gonads of all males from each dietary treatment were used for analysis of sperm motility (*N* = 27; nine fish/tank; *N* = 3).

### Histology

The livers were fixed in Bouin’s solution and dehydrated in ethanol, cleared in xylene, embedded in paraffin blocks, and cut with a rotary microtome (Leica, Bensheim, Germany) into 5-μm sections and then stained with hematoxylin and eosin using standard procedures. Histological observations were done under a light microscope (Nikon E600, Tokyo, Japan).

### COX enzyme activity

For the preparation of homogenates of liver tissue, samples were washed in Tris–HCl buffer (100 mM, pH 7.4) to remove the remnants of the surrounding tissue and blood. Then, they were homogenized in buffer (100 mM Tris–HCl, 1 mM EDTA, pH 7.8) in a volume of 5 ml/g liver, and the homogenate was then centrifuged for 15 min at 10,000*g* at 4 °C. Supernatant was frozen at −80 °C. The analysis of COX enzyme activity was determined using the COX Activity Assay Kit from Cayman Chemical, 1180 E. Ellsworth Rd., Ann Arbor, MI 48108, USA (Flippin et al. 2007).

COX activity was measured by the transformation of arachidonic acid to PGG2, determined indirectly by the quantitative conversion of PGG2 to PGH2 through use of the colorimetric substrate TMPD. To determine the activity of COX enzymes, inhibitors for both COX1 and COX2 enzymes (SC-560 and DuP-697, respectively) were used. The intensity of the color change reflected the amount of the reaction substrate TMPD consumed. After 5 min incubation at 25 °C, the sample was measured colorimetrically at a wavelength of 590 nm. COX activities were determined by subtracting the total activity (from samples without inhibitor) from the activity obtained from the samples with inhibitors. All tests were analyzed in duplicate.

### Cell-mediated immunity

To examine cell-mediated immunity, leukocytes were isolated from the spleens and pronephros of the fish. The organs were removed aseptically from the fish and pressed through a 60 μm nylon mesh into RPMI-1640 medium (Sigma-Aldrich, St. Louis, Missouri, USA) with l-glutamine and heparin (Biomed, Warsaw, Poland). The cell suspension was run on density gradients, Gradisol G (Aqua-Medica, Łódź, Poland) to isolate phagocytic cells or Gradisol L (Aqua-Medica, Łódź, Poland) to isolate lymphocytes, and then centrifuged at 400*g* for 40 min at 4 °C (Siwicki and Dunier 1990; Małaczewska et al. 2014).

The metabolic activity of spleen phagocytes was determined by measuring the intracellular respiratory burst activity (RBA) after stimulation with phorbol 12-myristate 13-acetate (PMA, Sigma-Aldrich, St. Louis, Missouri, USA) (Siwicki and Robak 2011). The method described by Siwicki and Robak (2011) was used to measure the potential killing activity (PKA) of spleen phagocytes.

The proliferative response of pronephric lymphocytes stimulated with the mitogen concanavalin A (ConA, Sigma, NY, USA) or lipopolysaccharide (LPS, Sigma, NY, USA) was determined using the MTT [3-(*4*,*5*-*dimethylthiazol*-*2*-*yl*)-2,5-diphenyltetrazolium bromide] (Sigma, NY, USA) colorimetric assay (Mosmann 1983), with modifications described for fish species by Siwicki and Robak (2011).

The percentage of phagocytising spleen macrophages and their phagocytic activity (number of bacteria absorbed by a single cell based on fluorescence intensity, see below) were determined using the Phagotest (Orpegen Pharma) procedure. All test reagents were prepared in accordance with the manufacturer’s recommendations. Cellular phagocytising activity was measured in a flow cytometer (Beckman Coulter, Epics XL) within 60 min after the last reagent had been added. The Phagotest was performed with the use of fluorescein-stained cells of *Escherichia coli*, which were phagocytised by macrophages. Cell nuclei were also stained. The results were analyzed with FlowJo software (version 10, Tree Star Inc., Stanford, CA, USA).

### Sperm motility

The testis was removed and transferred to 1.5-ml tubes contained 10 μl of Hanks’ balanced salt solution (HBSS; 0.137 M NaCl, 5.4 mM KCl, 1.3 mM CaCl_2_, 1.0 mM MgSO_4_, 0.25 mM Na_2_HPO_4_, 0.44 mM KH_2_PO_4_, 4.2 mM NaHCO_3_, and 5.55 mM glucose, pH 7.2) supplemented with 50 mM trehalose; bovine serum albumin (BSA), 0.5 %, was used as the extender. Sperm motility was observed 1 h after sample collection. Activated sperm was placed on a Teflon-coated 12-well slide (Tekdon, Inc., Myakka City, USA) and covered with a standard cover slip. A 1 μl mixture of sperm and Hanks’ balanced salt solution was placed on the slide, and sperm movement was recorded 6 s after activation. Video recordings for CASA (computer assay sperm analysis) were made using a microscope with a 20× negative phase objective. Recordings were made with a Basler a 202 K digital camera integrated with an Olympus BX51 microscope. The recording speed was 47 frames per second. The first 200 frames from each recording were analyzed using the program Image House, CRISMAS Company Ltd. From fifteen motility parameters, percentage of motile sperm (MOT,  %), curvilinear velocity (VCL, μm/s), amplitude of lateral head displacement (ALH, μm), average path velocity (VAP, μm/s), linearity (LIN = 100 × VSL/VCL,  %), beat-cross frequency (BCF, Hz) were chosen for further analysis.

### Data analysis

The results of all the measurements and calculations were subjected to statistical analysis with the GraphPad Prism program (Soft. Inc., Avenida de la Playa la Jolla, USA). Experimental results are reported as means (±SD). The means were analyzed with a one-way analysis of variance (ANOVA) and Tukey’s least significant difference test (*P* ≤ 0.05). For all calculations, *P* < 0.05 was considered as significant. All values were expressed as percentages prior to statistical processing and were transformed with *arcsin*.

## Results

### Effect of dietary resveratrol on cell-mediated immunity of medaka broodstock

**Fig. 1:**
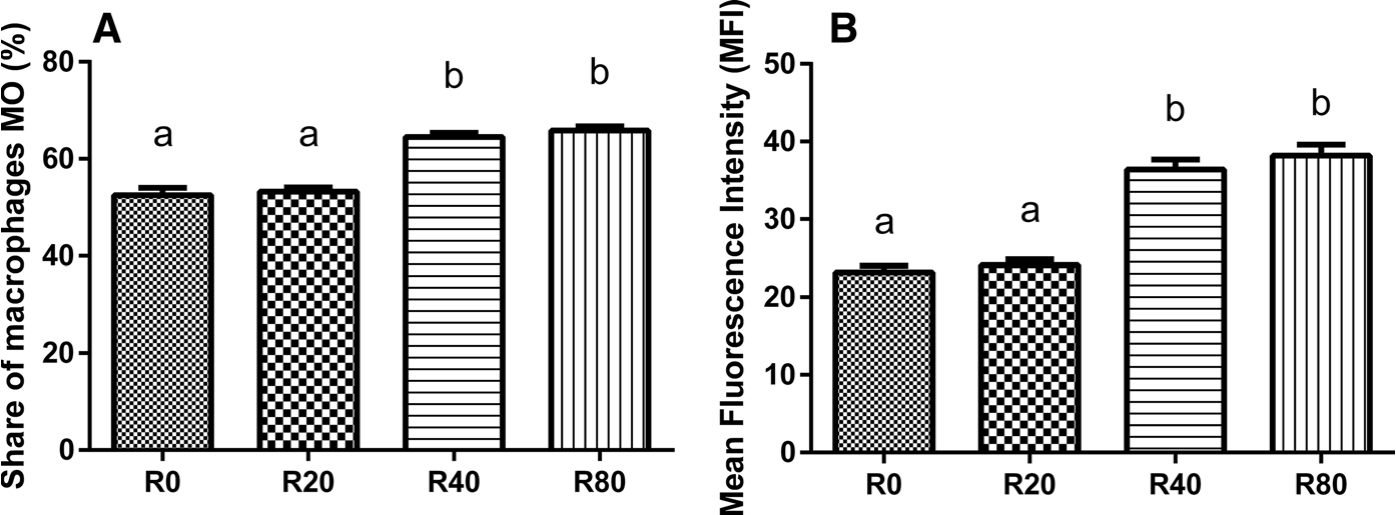
Percentage of macrophages (**a**) and mean fluorescence intensities (**b**) of medaka broodstock. R0, group fed control feed; R20, R40, and R80, groups fed feed with resveratrol at 20, 40, and 80 µg/g BW/day, respectively. Reported values are the mean ± SD. Mean values with *different superscripts* within a parameter column are significantly different (*P* < 0.05)

Fish fed on a diet supplemented with resveratrol at 40 and 80 µg/g BW/day (R40 and R80 groups) had significantly higher metabolic activity and intracellular phagocyte killing activity than control group (*P* < 0.05) (Table 1). The proliferative lymphocyte activity of the fish from R80 group was higher by more than 20 % in comparison with the control, R0 group (*P* < 0.05) (Table 1). Moreover, the percentage of macrophages (MO) and their mean fluorescence intensities (MFI) in R40 and R80 groups were significantly greater compared to C and R20 groups (*P* < 0.05) (Fig. 1). The differences in MO and MFI values ranged from 52.5 % (±1.5; R0 group) to 65.8 % (±1.6; R80 group) and from 23.2 (±1.4; R0 group) to 38.2 (±2.4; R80 group), respectively.

**Table 1:** Cell-mediated immunity in medaka fed experimental diets (mean ± SD; *N* = 45)

	Dietary treatments^a^			
	R0	R20	R40	R80
Metabolic ability of phagocytes (RBA; OD 620 nm)	0.40^a^ ± 0.04	0.43^a^ ± 0.03	0.46^b^ ± 0.04	0.50^b^ ± 0.05
Potential killing activity of phagocytes (PKA; OD 620 nm)	0.32^a^ ± 0.03	0.34^a^ ± 0.03	0.36^b^ ± 0.03	0.39^b^ ± 0.04
Lymphocyte proliferation stimulated by ConA (LP-ConA; OD 620 nm)	0.35^a^ ± 0.04	0.38^a^ ± 0.04	0.40^b^ ± 0.03	0.43^b^ ± 0.04
Lymphocyte proliferation stimulated by LPS (LP-LPS OD 620 nm)	0.27^a^ ± 0.03	0.29^a^ ± 0.04	0.32^b^ ± 0.04	0.36^b^ ± 0.04

Different superscript letters on the same line denote significant differences (*P* < 0.05)

^a^R0, group fed control feed; R20, R40, and R80, groups fed feed with resveratrol at 20, 40, and 80 µg/g BW/day

### Effect of dietary resveratrol on histology and liver COX activity

**Fig. 2:**
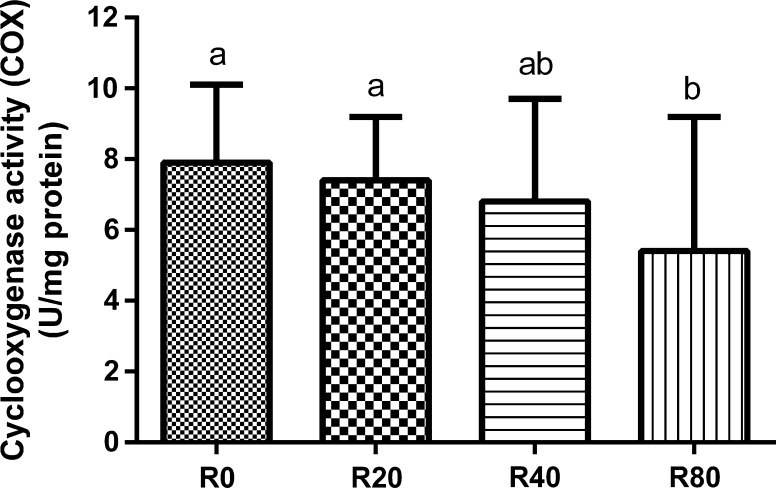
Cyclooxygenase activity (COX) measured in the females livers (mean ± SD; *N* = 15). R0, group fed control feed; R20, R40, and R80, groups fed feed with resveratrol at 20, 40, and 80 µg/g BW/day, respectively. Mean values with *different superscripts* within a parameter column are significantly different (*P* < 0.05)

The experimental diets had no effect on HSI values (*P* > 0.05; Table 2) as well as histological structures of the liver. Resveratrol at 80 µg/g BW/day decreased liver COX activity, i.e. 5.4 in R80 group and 7.9 in R0 group (*P* < 0.05; Fig. 2).

**Table 2:** Female hepatosomatic index (HSI), and egg quality obtained from broodstock fed experimental diets (mean ± SD; *N* = 3)

	Dietary treatment^a^			
	R0	R20	R40	R80
Hepatosomatic index HSI (%)	4.41 ± 1.12	3.98 ± 0.79	4.68 ± 1.17	4.70 ± 0.71
Fertility (pcs. egg/female)	7.90 ± 0.93	8.65 ± 3.43	9.93 ± 5.29	8.27 ± 3.47
Size of oocyte (mm)	1.24 ± 0.31	1.26 ± 0.28	1.23 ± 0.32	1.25 ± 0.08
Fertilized oocytes (%)	95.04 ± 1.79	94.35 ± 3.63	94.20 ± 0.16	92.53 ± 0.47
Embryo survival (%)	78.79^b^ ± 1.80	75.96^ab^ ± 9.51	67.22^a^ ± 7.62	71.54^ab^ ± 5.56
Hatching rate (%)	66.90^ab^ ± 14.94	59.58^a^ ± 11.37	90.43^c^ ± 15.81	77.39^bc^ ± 16.91

Different superscript letters on the same line denote significant differences (*P* < 0.05)

^a^R0, group fed control feed; R20, R40, and R80, groups fed feed with resveratrol at 20, 40 and 80 µg/g BW/day, respectively

### Effect of dietary resveratrol on sperm motility

**Fig. 3:**
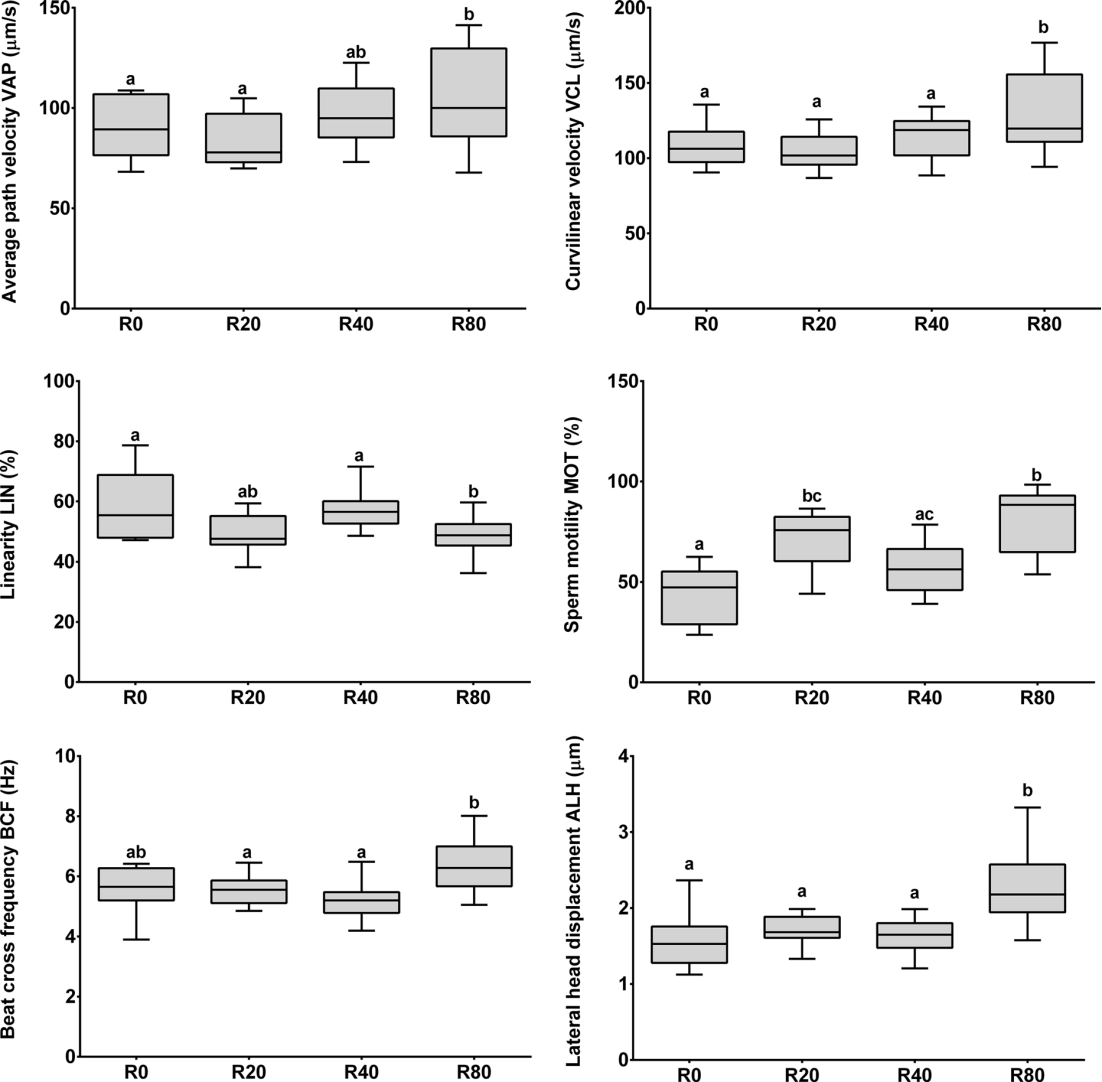
Comparison of sperm motility parameters of medaka after dietary exposure to resveratrol. R0, group fed control feed; R20, R40, and R80, groups fed feed with resveratrol at 20, 40, and 80 µg/g BW/day, respectively. Reported values are the mean ± SD. Mean values with *different superscripts* within a parameter column are significantly different (*P* < 0.05)

Supplementing the feed with resveratrol influenced sperm motility parameters (MOT, VCL, ALH, VAP, LIN, BCF) (*P* < 0.05) (Fig. 3). The percentage of motile sperm (MOT) ranged from 43.68 % in the control group to 80.33 % in R80 group (*P* < 0.05; Fig. 3). Sperm of males fed feed supplemented with resveratrol at 80 µg/g BW/day exhibited the highest curvilinear velocity (VCL) and greatest amplitude of lateral head displacement (ALH) compared to the rest of the dietary treatments (*P* < 0.05; Fig. 3). Consistent with this result, whereas VAP was highest in R80 group, linearity (LIN) was the lowest compared to the control (*P* < 0.05; Fig. 3).

### Effect of dietary resveratrol level on egg quality and offspring production

**Fig. 4:**
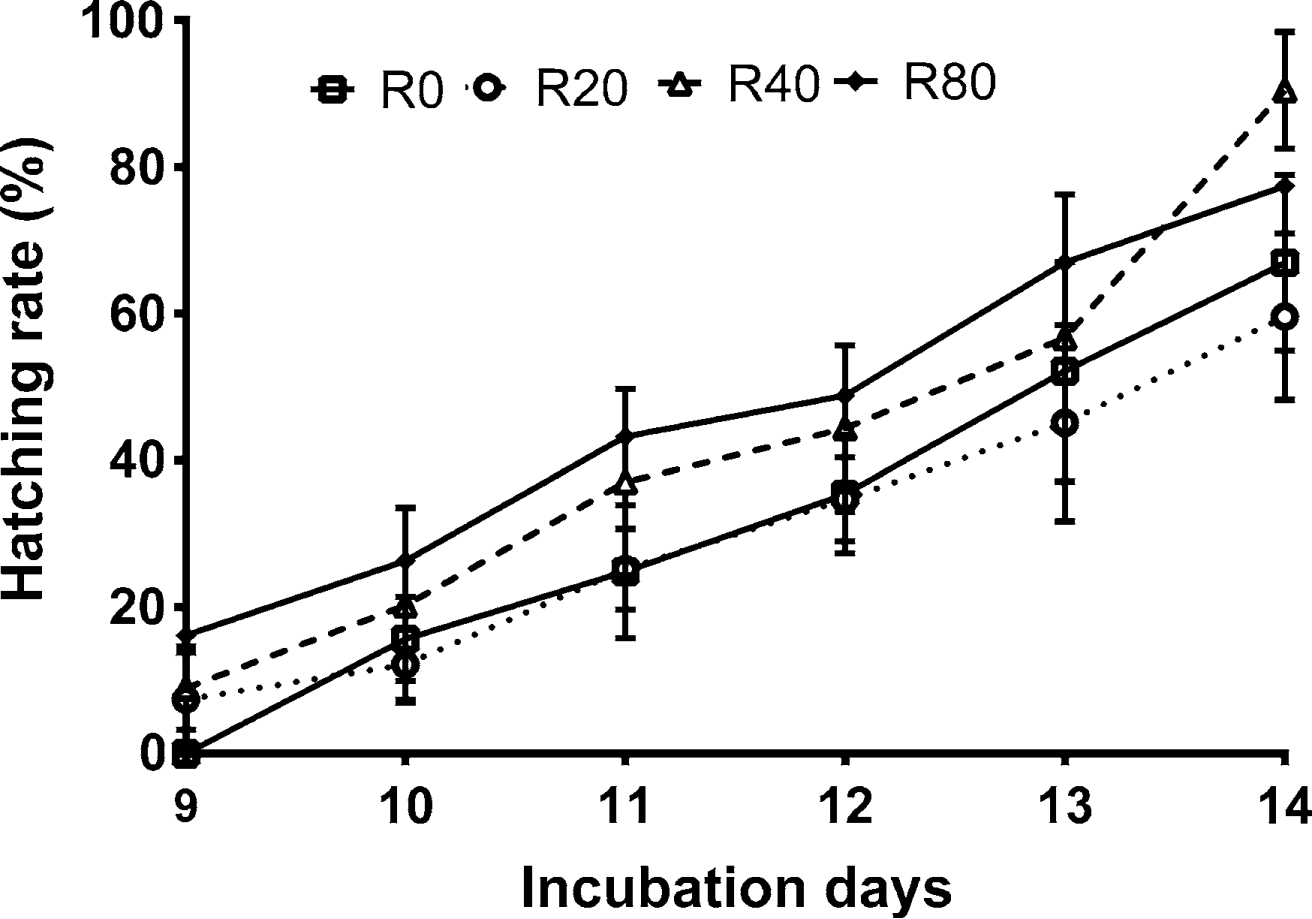
Percent of hatch derived from broodstock fed experimental diets. R0, group fed control feed; R20, R40, and R80, groups fed feed with resveratrol at 20, 40, and 80 µg/g BW/day, respectively

No differences were seen between the dietary treatments in the fertility of females (*P* > 0.05, Table 2). Oocytes ranged from 1.23 to 1.26 mm in diameter size (*P* > 0.05, Table 2). During the course of the experiment, the percentage of fertilized oocytes marginally decreased with increasing amounts of resveratrol in the feed (*P* > 0.05; Table 2). Initial embryo survival ranged from 79.02 % (±0.98) (R80 group) to 82.05 % (±1.26) (R40 group). Supplementing broodstock feed with resveratrol at 40 µg/g BW/day (R40 group) lowered the survival of embryos compared to the control, R0 group (*P* < 0.05; Table 2). Moreover, a statistically significant difference in hatch rate between dietary treatments was observed (Table 2, Fig. 4). Supplementation of the broodstock feed with resveratrol diet at 80 µg/g BW/day resulted in the highest offspring hatch rate during the nine days of egg incubation time (*P* < 0.05; Fig. 4). The hatch rate at 14 days ranged from 59.58 in R20 group to 90.43 in R40 group (*P* < 0.05; Table 2; Fig. 4).

## Discussion

The results of this study suggest that increasing the level of resveratrol in the diet has a beneficial effect on cell-mediated immunity in medaka. In mice, resveratrol promotes lymphocyte proliferation in a concentration-dependent manner and simultaneously induces the production of certain interleukins (Feng et al. 2002). Furthermore, the effect of resveratrol as an immunomodulator that inhibits synthesis and release of the pro-inflammatory mediator, PGE2, which is produced by monocytes and thrombocytes from arachidonic acid, has been recognized (Das and Das 2007; Chen et al. 2014). Moreover, resveratrol leads to a reduction in the production of reactive oxygen species in neutrophils and macrophages in mammals (Kohnen et al. 2007) and in leukocytes induced by PMA in fish (Castro et al. 2008). Phagocytes in fish (especially neutrophils) produce large amounts of reactive oxygen species (ROS) after stimulation. Elevated ROS production during the respiratory burst plays a protective role by killing pathogens, but at the same time, it induces oxidative stress (Reynolds et al. 2007). Decreasing the amounts of ROS in fish leukocytes by incubation with resveratrol has been found to have a ROS-scavenging, protective effect on internal organs (Castro et al. 2008). In other research, resveratrol added as a vaccine adjuvant does not affect serum antibody levels but inhibits the expression of genes involved in the inflammatory response, including the expression of genes in kidney leukocytes (Domínguez et al. 2013). Resveratrol blocks the inflammatory response, including cell migration, respiratory burst activity, and PG synthesis, and it has a strong inhibitory effect on the expression and activity of intra- and extracellular peroxidase activity in neutrophils (Castro et al. 2008). Our data suggest that resveratrol strongly modulates phagocyte and lymphocyte activity in medaka. Simultaneously, the positive effect of resveratrol at 80 μg/g BW on the medaka immune system caused some perturbance of normal reproduction during the spawning season.

In our studies, we found an inhibitory effect of resveratrol, at a dose of 80 μg/g BW, on COX activity. This result indicated that physiological expression and enzymatic activity COX occured in the liver, and that chemical agents could affect COX expression or activity during the spawning season. The gene expression of COX1, which dictates the synthesis of prostanoids and the proper function of internal organs, is fixed and characteristic for most cells of higher vertebrates (Dannhardt and Kiefer 2001). Resveratrol in the broodstock diet increased lymphocyte proliferation, and this increase might lead to immunosuppression of the expression of COX in the medaka ovary. In fish production of interleukins in macrophage culture showed a down-regulation when PGE2 increase, and interleukins are able to stimulate PGs production by the induction of COX2 activity (Furne et al. 2013). The commonness of COX isoenzymes in tissues and the high activity and crucial importance of prostanoids in fish oogenesis (Sorbera et al. 2001) lead us to conclude that resveratrol in the medaka diet might contributes to negative effect on the reproduction.

The effect on hepatic growth, but with another COX inhibitor, ibuprofen, in the medaka female diet was shown by Flippin et al. (2007), who showed that this relationship applies only to females and results from effects on vitellogenin synthesis in the liver. The impact of COX on increased estrogen synthesis in mammals (Egan et al. 2004), its influence on vitellogenin synthesis in fish (Verslycke et al. 2002), and its effects even in medaka males (Hashimoto et al. 2009), all suggest that exposure to resveratrol contributes in a dose- and time-dependent manner to vitellogenesis and, consequently, to the maturation of oocytes in medaka (Seki et al. 2002). A previous study has shown that a dose of 20 mg/g BW disrupted oogenesis in the medaka ovary (Kowalska et al. 2011). However, although vitellogenin production can raise HSI values (Pereira et al. 1993), in the current study, the HSI values in females were similar in all treatments. However, the noted differences in reproduction might have resulted from the effect of resveratrol on other functions, i.e. fatty acid synthesis, glycogen storage, metabolism of eicosanoids, and bioconversion of ARA, as well as detoxification of contaminants in the liver (Gravel and Vijayan 2006). Triebskorn et al. (2004) observed that COX inhibitors can reduce the amount of glycogen granules in hepatocytes. The higher doses of resveratrol used in this study, in R40 and R80 groups, could disrupt the synthesis of vitellogenin, a glycoprotein precursor to yolk (Lazier and MacKay 1993), or perturb the bioconversion to eicosanoids, especially from ARA, which influences the survival of embryos (Kowalska and Kowalski 2014).

In our study, survival was lowest for embryos from females with lower liver COX activity. Similarly, disruption of COX1 expression disrupts the development of embryos of zebrafish *Danio rerio* (Grosser et al. 2006). It can be concluded that dietary resveratrol in medaka diet (40–80 μg/g BW) alters COX activity in the tissues, prostaglandin synthesis, and the balance between them. Prostaglandin E2 (PGE2) was identified as an important PGE in the medaka ovary (Fujimori et al. 2011). In zebrafish, specific receptors dependent on prostaglandin PGE2 play an essential role in gastrulation during embryogenesis (Cha et al. 2006). In addition, an increase in the cellular immune response, induced at doses of resveratrol of 40 and 80 mg/g BW in the feed, could also indirectly inhibit COX2 gene expression in the ovaries of females. In mammals, the isoform of this enzyme (induced, COX2) is also responsible for the development of embryos (Burdan 2005).

The presence of resveratrol at 80 μg/g BW in diets negatively affected several sperm motility parameters (VCL, VAP, BCF, ALH, and LIN). In males, the negative impact of even low doses of resveratrol is manifested as a slight deterioration in the quality of sperm during the spawning season (Kowalska et al. 2011). The role of resveratrol in male fish reproduction is still not well defined, even though its effect on COX activity has been confirmed (Castro et al. 2008; Fujimori et al. 2011; this study). In medaka, short-term exposure (3 weeks) to certain chemicals, e.g. synthetic estrogen, affects sperm swimming speed (Hashimoto et al. 2009), but just as we show here, it does not affect the percentage of motile spermatozoa or the fertilization rate. An earlier study has shown that the amount of ARA in the diet, not the addition of resveratrol (dose tested 20 μg/g BW), determines the quantity of fertilized oocytes (Kowalska and Kowalski 2014).

The role of resveratrol in fish physiology and reproduction is not fully understood. It is known that resveratrol enhances the immune response, but as shown here, some reproductive parameters in females and males can be negatively impacted. Interestingly, resveratrol has been shown to increase the lifespan of a fish in a dose-dependent manner (Valenzano et al. 2006). This ability might relate to a reduction in reproductive effort, as reflected by lower gamete quality (seen as lower sperm motility and reduced embryo survival, this study). Resveratrol is not only an inhibitor, but also an antioxidant, as tested in high doses (Valenzano et al. 2006; this study) probably had other effects. The high hatching rate in R40 group (this study) could be related to the effects of resveratrol on induction of the immune system and its inhibitory effect on the production of reactive oxygen species (Castro et al. 2008). As shown here, resveratrol as a polyphenol immunomodulator has beneficial effects on fish but as a COX inhibitor may cause a failure in reproduction of broodstock.

## Conclusion

Our results showed that high resveratrol inhibits reproductive performance so its potential as a feed additive for fish broodstock is inappropriate. The results indicate that a diet supplemented with resveratrol at a dosage of 40 μg/g BW improves phagocyte killing ability and lymphocyte proliferation in broodstock and accelerates offspring hatch. Also, the results suggest that COX activity influences sperm and oocyte quality in fish; the presence of a COX inhibitor in the dose of 40 μg/g decreased the embryo survival.

## References

[CR1] Ashley PJ (2007). Fish welfare: current issues in aquaculture. Appl Anim Behav Sci.

[CR2] Barton BA, Iwama GK (1991). Physiological changes in fish from stress in aquaculture with emphasis on the response and effects of corticosteroids. Annu Rev Fish Dis.

[CR3] Burdan F (2005). Comparison of developmental toxicity of selective and nonselective cyclooxygenase-2 inhibitors in CRL: (WI)WUBR Wistar rats—DFU and piroxicam study. Toxicology.

[CR4] Castro R, Lamas J, Morais P, Sanmartín ML, Orallo F, Leiro J (2008). Resveratrol modulates innate and inflammatory responses in fish leucocytes. Vet Immunol Immunopathol.

[CR5] Cha YI, Kim SH, Sepich D, Buchanan FG, Solnica-Krezel L, DuBois RN (2006). Cyclooxygenase-1-derived PGE2 promotes cell motility via the G-protein-coupled EP4 receptor during vertebrate gastrulation. Genes Dev.

[CR6] Chen X, Lu J, An M, Ma Z, Zong H, Yang J (2014). Anti-inflammatory effect of resveratrol on adjuvant arthritis rats with abnormal immunological function via the reduction of cyclooxygenase-2 and prostaglandin E2. Mol Med Rep.

[CR7] Dannhardt G, Kiefer W (2001). Cyclooxygenase inhibitors–current status and future prospects. Eur J Med Chem.

[CR8] Das S, Das DK (2007). Anti-inflammatory responses of resveratrol. Inflamm Allergy Drug Targets.

[CR9] Domínguez B, Noia M, Leiro J, Lamas J (2013). Regulation by resveratrol of turbot inflammatory response induced by vaccines. Fish Shellfish Immunol.

[CR10] Egan KM, Lawson JA, Fries S, Koller B, Rader DJ, Smyth EM, Fitzgerald GA (2004). COX-2 derived prostacyclin confers atheroprotection on female mice. Science.

[CR11] Feng YH, Zhou WL, Wu QL, Li XY, Zhao WM, Zou JP (2002). Low dose of resveratrol enhanced immune response of mice. Acta Pharmacol Sin.

[CR12] Flippin JL, Huggett D, Foran CM (2007). Changes in the timing of reproduction following chronic exposure to ibuprofen in Japanese medaka, *Oryzias latipes*. Aquat Toxicol.

[CR13] Fujimori Ch, Ogiwara K, Hagiwara A, Rajapakse S, Kimura A, Takahashi T (2011). Expression of cyclooxygenase-2 and prostaglandin receptor EP4b mRNA in the ovary of the medaka fish, *Oryzias latipes*: possible involvement in ovulation. Mol Cell Endocrinol.

[CR14] Furne M, Holen E, Araujo P, Lie K, Moren M (2013). Cytokine gene expression and prostaglandin production in head kidney leukocytes isolated from Atlantic cod (*Gadus morhua*) added different levels of arachidonic acid and eicosapentaenoic acid. Fish Shellfish Immunol.

[CR15] Gravel A, Vijayan MM (2006). Salicylate disrupts interrenal steroidogenesis and brain glucocorticoid receptor expression in rainbow trout. Toxicol Sci.

[CR16] Grosser T, Fries S, Fitzgerald GA (2006). Biological basis for the cardiovascular consequences of COX-2 inhibition: theraqpeutic challenges and opportunities. J Clin Invest.

[CR17] Hashimoto S, Watanabe E, Ikeda M, Terao Y, Strüssmann CA, Inoue M, Hara A (2009). Effects of ethinylestradiol on medaka (*Oryzias latipes*) as measured by sperm motility and fertilization success. Arch Environ Contam Toxicol.

[CR18] Hata AN, Breyer RM (2004). Pharmacology and signaling of prostaglandin receptors: multiple roles in inflammation and immune modulation. Pharmacol Ther.

[CR19] Hong HN, Kim HN, Park KS, Lee SK, Gu MB (2007). Analysis of the effects diclofenac has on Japanese medaka (*Oryzias latipes*) using real-time PCR. Chemosphere.

[CR20] Ishikawa TO, Herschman HR (2007). Two inducible, functional cyclooxygenase-2 genes are present in the rainbow trout genome. J Cell Biochem.

[CR21] Ishikawa TO, Griffin KJP, Banerjee U, Herschman HR (2007). The zebrafish genome contains two inducible, functional cyclooxygenase-2 genes. Biochem Biophys Res Commun.

[CR22] Kinoshita M, Murata K, Naruse K, Tanaka M, Kinoshita M, Murata K, Naruse K, Tanaka M (2009). Medaka management. Medaka. biology, management, and experimental protocols.

[CR23] Kohnen S, Franck T, Van Antwerpen P, Boudjeltia KZ, Mouithys-Mickalad A, Deby C, Moguilevsky N, Deby-Dupont G, Lamy M, Serteyn D (2007). Resveratrol inhibits the activity of equine neutrophil myeloperoxidase by a direct interaction with the enzyme. J Agric Food Chem.

[CR24] Kowalska A, Kowalski R (2014). The effect of cyclooksygenase (COX) inhibitors on Japanese medaka (*Oryzias latipes*) reproduction parameters fed with high level of arachidonic acid (20:4 n-6). Aquac Int.

[CR25] Kowalska A, Kowalski R, Zakęś Z (2011). The effect of selective cyclooxygenase (COX) inhibitors on Japanese medaka (*Oryzias latipes*) reproduction parameters. World Acad Sci Eng Technol.

[CR26] Lazier CB, MacKay ME, Hochachka PW, Mommsen TP (1993). Vitellogenin gene expression in teleost fish. Biochemistry and molecular biology of fishes.

[CR27] Liu W, Cao D, Oh SF, Serhan CN, Kulmacz RJ (2006). Divergent cyclooxygenase responses to fatty acid structure and peroxide level in fish and mammalian prostaglandin H synthases. FASEB J.

[CR28] Magnadóttir B (2006). Innate immunity of fish (overview). Fish Shellfish Immunol.

[CR29] Małaczewska J, Siwicki AK, Wójcik R, Turski WA, Kaczorek E (2014). The in vitro effect of kynurenic acid on the rainbow trout (*Oncorhynchus mykiss*) leukocyte and splenocyte activity. Pol J Vet Sci.

[CR30] Martinez J, Moreno JJ (2000). Effect of resveratrol, a natural polyphenolic compound, on reactive oxygen species and prostaglandin production. Biochem Pharmacol.

[CR31] Minghetti L, Polazzi E, Nicolini A, Créminon C, Levi G (1997). Up-regulation of cyclooxygenase-2 expression in cultured microglia by prostaglandin E2, cyclic AMP and non-steroidal anti-inflammatory drugs. Eur J Neurosci.

[CR32] Mosmann T (1983). Rapidcolorimetric assay for cellular growth and survival: application to proliferation and cytotoxicity assays. J Immunol Methods.

[CR33] Oliva-Teles A (2012). Nutrition and health of aquaculture fish. J Fish Dis.

[CR34] Pereira JJ, Mercado-Allen R, Kuropat C, Luedke D, Sennefelder G (1993). Effect of cadium ccumulation on serum vitellogenin levels and hepatosomatic and gonadosomatic indices of winter flounder (*Pleuronectes americanus*). Arch Environ Contam Toxicol.

[CR35] Reynolds A, Laurie C, Mosley RL, Gendelman HE (2007). Oxidative stress and the pathogenesis of neurodegenerative disorders. Int Rev Neurobiol.

[CR36] Rowley AF, Knight J, Lloyd-Evans P, Holland JW, Vickers PJ (1995). Eicosanoids and their role in immune modulation in fish—a brief overview. Fish Shellfish Immunol.

[CR37] Sahin K, Orhan C, Akdemir F, Tuzcu M, Iben C, Sahin N (2012). Resveratrol protects quail hepatocytes against heat stress: modulation of the Nrf2 transcription factor and heat shock proteins. J Anim Physiol Anim Nutr.

[CR38] Seki M, Yokota H, Matsubara H, Tsuruda Y, Maeda M, Tadokoro H, Kobayashi K (2002). Effect of ethinylestradiol on the reproduction and induction of vitellogenin and testis-ova in medaka (*Oryzias latipes*). Environ Toxicol Chem.

[CR39] Siwicki AK, Dunier M (1990). Effect of levamisole on the lymphocyte and macrophage activity in carp (*Cyprinus carpio*). Ann Rech Vet.

[CR40] Siwicki AK, Robak S (2011). The innate immunity of European eel (*Anguilla anguilla*) growing in natural conditions and intensive system of rearing. Centr Eur J Immunol.

[CR41] Siwicki AK, Anderson DP, Studnicka M (1994). The immune system of fish. Arch Pol Fish.

[CR42] Sorbera LA, Asturiano JF, Carillo M, Zanuy S (2001). Effects of polyunsaturated fatty acids and prostaglandins on oocyte maturation in a marine teleost, the European seabass (*Dicentrarchus labrax*). Biol Reprod.

[CR43] Stacey NE, Goetz FW (1982). Role of prostaglandins in fish reproduction. Can J Fish Aquat Sci.

[CR44] Triebskorn R, Casper H, Heyd A, Eikemper R, Köhler HR, Schwaiger J (2004). Toxic effects of the non-steroidal anti-inflammatory drug diclofenac. Part II: cytological effects in liver, kidney, gills and intestine of rainbow trout (*Oncorhynchus mykiss*). Aquat Toxicol.

[CR45] Valenzano DR, Terzibasi E, Genade T, Cattaneo A, Domenici L, Cellerino A (2006). Resveratrol prolongs lifespan and retards the onset of age-related markers in a short-lived vertebrate. Curr Biol.

[CR46] Verslycke T, Vandenbergh GF, Versonnen BJ, Arijs K, Janssen CR (2002). Induction of vitellogenesis in 17a-ethinylestradiolexposed rainbow trout (*Oncorhynchus mykiss*): a method comparison. Comp Biochem Physiol C: Toxicol Pharmacol.

[CR47] Wedemeyer GA, Iwama GK, Pickering AD, Sumpter JP, Schreck CB (1997). Effect of rearing conditions on the health and physiological quality of fish in intensive culture. Fish stress and health in aquaculture.

[CR48] Yamamoto T, Yamamoto T (1975). Introductory remarks on the medaka. Medaka (killifish): biology and strains.

